# The Crystal Structure of Human IgD‐Fc Reveals Unexpected Differences With Other Antibody Isotypes

**DOI:** 10.1002/prot.26771

**Published:** 2024-11-25

**Authors:** Anna M. Davies, Tam T. T. Bui, Raúl Pacheco‐Gómez, Susan K. Vester, Andrew J. Beavil, Hannah J. Gould, Brian J. Sutton, James M. McDonnell

**Affiliations:** ^1^ Randall Centre for Cell and Molecular Biophysics King's College London London UK; ^2^ Centre for Biomolecular Spectroscopy King's College London London UK; ^3^ Malvern Panalytical Ltd Malvern UK

**Keywords:** antibody, IgD, immune system, immunoglobulin, isotype

## Abstract

Of the five human antibody isotypes, the function of IgD is the least well‐understood, although various studies point to a role for IgD in mucosal immunity. IgD is also the least well structurally characterized isotype. Until recently, when crystal structures were reported for the IgD Fab, the only structural information available was a model for intact IgD based on solution scattering data. We now report the crystal structure of human IgD‐Fc solved at 3.0 Å resolution. Although similar in overall architecture to other human isotypes, IgD‐Fc displays markedly different orientations of the Cδ3 domains in the Cδ3 domain dimer and the lowest interface area of all the human isotypes. The nature of the residues that form the dimer interface also differs from those conserved in the other isotypes. By contrast, the interface between the Cδ2 and Cδ3 domains in each chain is the largest among the human isotypes. This interface is characterized by two binding pockets, not seen in other isotypes, and points to a potential role for the Cδ2/Cδ3 interface in stabilizing the IgD‐Fc homodimer. We investigated the thermal stability of IgD‐Fc, alone and in the context of an intact IgD antibody, and found that IgD‐Fc unfolds in a single transition. Human IgD‐Fc clearly has unique structural features not seen in the other human isotypes, and comparison with other mammalian IgD sequences suggests that these unique features might be widely conserved.

## 1 Introduction

Humans produce five antibody isotypes (IgA, IgD, IgE, IgG, and IgM) as part of the adaptive immune response, each with different effector functions [1]. IgD is the least well‐understood in terms of both its structure and function. Recent evidence has pointed to a role for IgD in mucosal immunity. In humans, 20%–25% of B cells in the upper respiratory mucosa are IgD^+^IgM^−^ and secrete IgD [2]. Secreted IgD has been shown to recognize food proteins and respiratory bacteria and to bind and activate basophils in a manner that is proinflammatory but does not induce degranulation [2, 3].

Despite the wealth of structural data generated for the four better‐characterized human isotypes, the only information that was available for IgD for nearly two decades was a model from X‐ray scattering studies that revealed a predominantly T‐shaped structure in solution [4]. Recently, we reported crystal structures of the IgD Fab, providing the first high resolution structural insights into this antibody isotype [5].

We now report the crystal structure of human IgD‐Fc, solved at 3.0 Å resolution. The structure is surprisingly different to the other human isotypes, including a unique arrangement of domains within the Cδ3 dimer. Furthermore, a uniquely extensive interface between the Cδ2 and Cδ3 domains within each chain may contribute to the stability of the IgD‐Fc homodimer. Sequence comparisons suggest that these unique features of human IgD are widely conserved between other mammalian species.

## 2 Materials and Methods

### 2.1 Protein Expression and Purification

The human IgD Fab and intact human IgD were expressed and purified as described previously [5, 6].

The human IgD‐Fc sequence was obtained from UniProt (accession number P01880: secreted IgD isoform 1). A C‐terminally His‐tagged IgD‐Fc construct (Lys286‐Lys513), which included a small number of residues from the lower hinge region (Figure S1), was synthesized commercially, cloned into a pcDNA3.4 vector, and used to transfect Expi293 HEK cells according to the manufacturer's instructions. The Expi293 HEK cells were then adapted to adherent growth conditions [7] in 24‐well plates and selected with geneticin; cultures of resistant cells were later expanded in flasks. Supernatants were screened for the presence of His‐tagged IgD‐Fc by surface plasmon resonance using an anti‐His‐tag chip.

Cultures for supernatants that exhibited the highest number of response units after capture onto the chip surface were expanded and adapted to growth in spinner flasks at 37°C and 8% CO_2_ in FreeStyle 293 Expression Medium and 600–700 μg/mL geneticin.

Cells were grown in 1 L spinner flasks for 1 month, after which supernatants were harvested, centrifuged at 4000 rpm, and then filtered using a 0.2 μm filter. The pH of supernatant was adjusted to 7.4. His‐tagged IgD‐Fc was purified from the supernatant using a His GraviTrap Talon column (Cytiva). Protein was eluted from the column with a buffer comprising 50 mM sodium phosphate, 300 mM sodium chloride, and 200 mM imidazole, pH 7.4. Eluted protein was concentrated, further purified by size exclusion chromatography using a Superdex 200 10/300 GL column (Cytiva) and eluted in PBS pH 7.4 containing 0.1% (w/v) sodium azide.

### 2.2 Crystallization

Recombinant human IgD‐Fc was concentrated to 5.1 mg/mL. Crystals were grown at 18°C in SWISSCI MRC 96‐well plates. The reservoir comprised 100 μL of 0.1 M Bis‐Tris pH 5.5, 25% (w/v) PEG 3350, and 0.2 M ammonium acetate. The drops comprised 100 nL protein solution and 100 nL reservoir. Crystals grew as thin plates and were cryoprotected with 0.1 M sodium acetate pH 5.5, 35% (w/v) PEG 3350, and 17% (v/v) ethylene glycol before flash‐cooling in liquid nitrogen.

### 2.3 Structure Determination, Model Building, and Refinement

X‐ray diffraction data were collected at beamline I24 at the Diamond Light Source (Harwell, UK). Data were integrated using the DIALS automatic processing pipeline at Diamond [8]. Data from four isomorphous crystals grown and cryoprotected under the same conditions were merged and further processed using programs from the CCP4 suite [9, 10]. The structure was solved by molecular replacement with PHASER [11] using a model of the Cδ2 domain predicted by AlphaFold [12] and Cγ3 domain atoms from PDB: 4C54 [13] as search models. Refinement was performed with PHENIX [14] and interactive model building with Coot [15]. Data processing and refinement statistics are summarized in Table 1. Figures were produced with PyMOL (The PyMOL Molecular Graphics System, Version 1.1r1, Schrödinger, LLC).

**TABLE 1:** Data processing and refinement statistics.

	IgD‐Fc
Data processing	
Space group	*P* 1 2_1_ 1
*a, b, c* (Å)	105.82, 43.67, 121.47
α, β, γ (°)	90.00, 107.96, 90.00
Resolution (Å)^a^	52.45–3.00 (3.18–3.00)
Completeness (%)^a^	99.9 (99.3)
No. of unique reflections^a^	21 769 (3452)
Multiplicity^a^	15.5 (15.8)
Mean (*I*)/*σ*(*I*)^a^	5.9 (2.0)
CC_1/2_ ^a^	0.962 (0.349)
*R*_merge_ (%)^a^	54.9 (196.1)
*R*_meas_ (%)^a^	56.7 (202.6)
*R*_pim_ (%)^a^	14.2 (50.4)
Wilson *B* factor (Å^2^)	38.73
Refinement	
*R*_work_/*R*_free_ (%)^b^	22.83 / 28.82
No. of reflections	21 745
No. of TLS groups	33
RMSD	
Bond lengths (Å)	0.003
Bond angles (°)	0.670
Coordinate error (Å)	0.51
No. of atoms	
Protein	5845
Solvent	15
Carbohydrate	407
Acetate	20
Ethylene glycol	8
Polyethylene glycol	7
Average *B* factor (Å^2^)	
Protein	41.24
Solvent	27.35
Carbohydrate	47.05
	42.1
Acetate	42.57
Ethylene glycol	47.89
Polyethylene glycol	34.46
Ramachandran plot	
Favored (%)	96.76
Allowed (%)	2.84
Outliers (%)	0.41
Rotamer outliers (%)	3.49

^a^ Values in parentheses are for the outer shell.

^b^ *R*_free_ set comprises 5% of reflections.

The boundaries defined by Halaby et al. [16] for the β‐strands and loops within the immunoglobulin fold were used as a guide for β‐strand and loop assignment. The complete backbone for the Cδ2 domain (residues Leu297‐Leu393) was modeled in all four chains. The Cδ3 domain (residues Pro401‐Leu501) was modeled as follows: Pro401‐Ser411, Ala418‐Pro454, Arg459‐Pro474, and Gln478‐Leu501 in Chain A, Pro401‐Ser411, Ala417‐Pro454, Thr461‐Pro472, and Ala480‐Ser500 in Chain B, Pro401‐Ala410, Ala417‐Pro455, Arg459‐Ala473, and Ala480‐Leu501 in Chain C, and Pro401‐Asp413, Glu416‐Pro454, Ser460‐Pro472, and Ala480‐Arg499 in Chain D.

Interfaces were analyzed with PISA [17]. For the analysis of the interfaces between CH2/CH3 and CH3/CH4 domains, any disordered residues were modeled in Coot [15] using standard rotamers.

### 2.4 Sequence Analysis

Sequences for human IgA1, IgD, IgE, IgG1, and IgM were obtained from UniProt [18] with the following accession numbers: IgA1, P01876; IgD, P01880; IgE, P01854; IgG1, P01857, and IgM, P01871. Mammalian IgD sequences were obtained from GenBank [19] with the following accession numbers: baboon, ABB89458; chimpanzee, DQ297174; cow, AF411240; cynomolgus monkey, ABB89460; dog, ABB89467; ferret, QJY40730; goat, AMP34156; gorilla, DBA12041; grivet, WEL32150; horse, AAU09793; mangabey, ABB89465; mole‐rat, KFO35301; mouse, AAB59654; orangutan, PNJ04969; panda, AAX73311; pig, BAI82567; rat, AAO19643; rhesus macaque, DQ297179, and sheep, AAN03671. Sequence alignments were performed with Clustal Omega [20].

### 2.5 Differential Scanning Calorimetry

Differential scanning calorimetry (DSC) was performed using a MicroCal PEAQ‐DSC instrument (Malvern Panalytical). Prior to the DSC experiments, proteins were purified by size exclusion chromatography and eluted in a buffer comprising PBS pH 7.4 and 0.1% (w/v) sodium azide. The protein concentrations were as follows: IgD‐Fc, 17.5 and 43.9 μM; IgD Fab, 23.9 μM, and intact IgD, 15.1 μM. Experiments were performed at a scan rate of 60°C/h over ranges of 10°C–110°C (IgD‐Fc at 17.5 μM) and 15°C–105°C (IgD‐Fc at 43.9 μM, IgD Fab at 23.9 μM and intact IgD at 15.11 μM). DSC data were baseline corrected and fitted using the MicroCal PEAQ‐DSC Software (Malvern Panalytical) and plotted with Origin 7 (OriginLab Corporation, Northampton, MA, USA).

## 3 Results

### 3.1 Overall Structure

**FIGURE 1:**
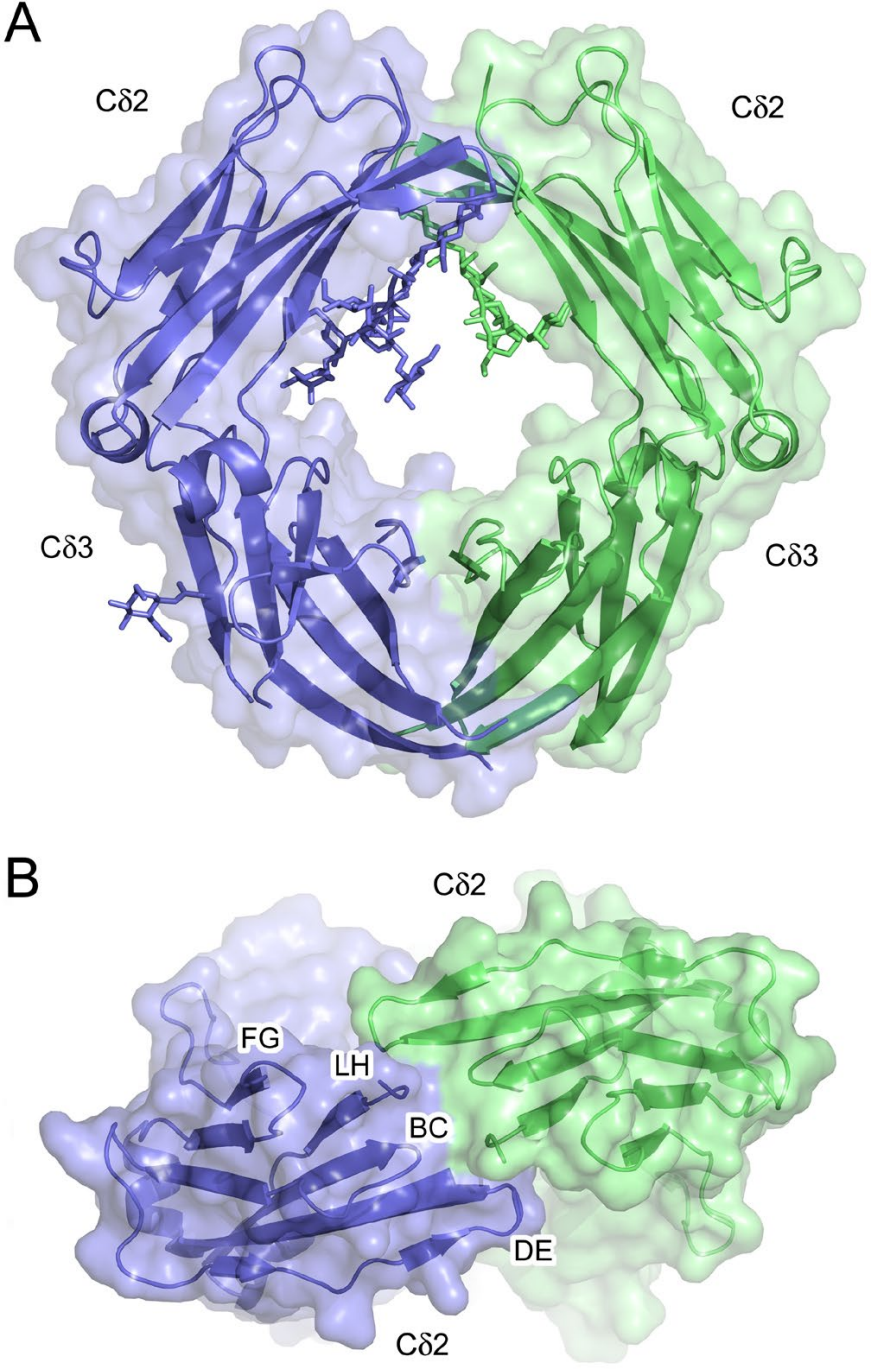
Overall structure of human IgD‐Fc. (A) Overall structure of the human IgD‐Fc homodimer, with the two Chains, C and D, colored green and blue, respectively. Oligosaccharide residues that are covalently attached to the Cδ2 and Cδ3 domains are shown using a sticks representation. (B) View of human IgD‐Fc to show the interface between the Cδ2 domains. The lower hinge (LH) and Cδ2 BC, DE, and FG loops are labeled.

We solved the crystal structure of human IgD‐Fc, with two molecules in the asymmetric unit, at 3.0 Å resolution. In the structure, molecule one comprises Chains A and B and molecule two comprises Chains C and D. IgD‐Fc is a homodimer (Figure 1A,B) and while the overall domain architecture resembles that for IgG‐ and IgA‐Fc, and the Cε3‐4 and Cμ3‐4 regions of IgE and IgM, respectively [21, 22, 23, 24], there are substantial differences in the disposition of the domains (Figure S2) that will be discussed in more detail below. The position of the Cδ2 domain relative to the Cδ3 domain is similar in each chain, and in each IgD‐Fc molecule, the Cδ2 domains contact one another, and the BC and DE loops and junction with the lower hinge form an interface that buries a surface area of ~255 Å^2^ (Figure 1B). Of the other human Fc regions, and with the exception of the Cε2 and Cμ2 domain interfaces in IgE and IgM, respectively, only the Cα2 domains in IgA contact one another, but the ~145 Å^2^ interface, which includes an interchain disulfide bond, is significantly lower than that found between the Cδ2 domains in IgD. The electrostatic surface potential of IgD‐Fc was compared to those for the other human isotypes (Figure S3). As expected, there is variation between the isotypes, but at a global level, no striking differences were observed for IgD.

### 3.2 Cδ2 Domain Structure and Glycosylation

**FIGURE 2:**
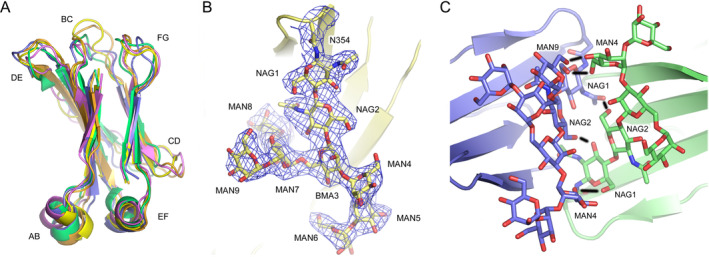
Overall structure of the Cδ2 domain. (A) With the exception of the flexible loop regions, the overall structure of the Cδ2 domain (Chain C, blue) is similar to that for Cα2 (IgA, green), Cε3 (IgE, orange), Cγ2 (IgG, yellow), and Cμ3 (IgM, purple). The following structures were used to generate the figure: IgA, PDB: 1OW0 [21]; IgE, PDB: 5MOL [22]; IgG, PDB: 1L6X [23]; IgM, PDB: 8BPG [24]. (B) Electron density for the oligosaccharide moiety in IgD‐Fc (Chain B). A 2F_o_ − F_c_ map is shown, contoured at 1*σ*. (C) Interaction between oligosaccharide residues in Chains C (green) and D (blue) of the human IgD‐Fc structure. Hydrogen bonds are depicted by black lines. In panels (B and C), oligosaccharide residues are labeled as follows: BMA, β‐D‐mannose; NAG, N‐acetylglucosamine; MAN, α‐D‐mannose.

The complete backbone for the Cδ2 domain (Leu297‐Leu393) was modeled in all four chains and the loop regions adopt similar conformations in each chain. The overall structure of the Cδ2 domain is similar to that for the human Cα2, Cγ2, Cε3, and Cμ3 domains (Figure 2). The Cδ2 AB, EF, and FG loops adopt similar conformations to those in the other isotypes, and the DE loop, to which the oligosaccharide moiety is covalently attached, is similar to the DE loop in IgE, IgG, and IgM. The BC and CD loops display greater conformational diversity.

**FIGURE 3:**
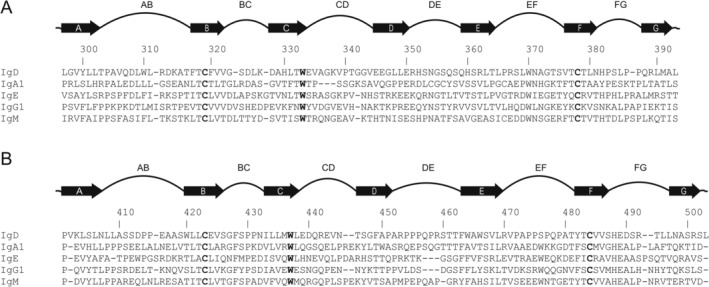
Sequence alignment of human immunoglobulin Fc constant domain sequences. Sequences were obtained from UniProt [18] with the following accession numbers: IgA1, P01876; IgD, P01880; IgE, P01854; IgG1, P01857; and IgM, P01871. The numbering scheme used is for human IgD. The sequence alignments were performed with Clustal Omega [20] and manual adjustments were made to ensure better agreement between structurally equivalent residues. (A) Sequence alignment of human Cδ2, Cα2, Cε3, Cγ2, and Cμ3 domains. (B) Sequence alignment of human Cδ3, Cα3, Cε4, Cγ3, and Cμ4 domains. The Cε2 and Cμ2 domains have not been included in the sequence alignment. In panels (A and B), the conserved amino acids of the “central pin” residues of immunoglobulin domains [58] are in bold.

The C‐terminal part of β‐strand G, which leads into the Cδ2–Cδ3 domain linker (Arg394‐Ala400), adopts a different conformation in IgD compared with the other CH2 (IgA and IgG) and CH3 (IgE and IgM) domains. In IgD, phenylalanine is found at position 317 (β‐strand B), compared with smaller aliphatic residues in the other isotypes (Figure 3). Alanine is found at position 392 (β‐strand G) and the sidechain of Trp370 (EF loop), a residue that is conserved in all isotypes, adopts a different position to avoid steric clashes with the bulkier phenylalanine (Figure S4). A conformational change occurs around the Ala392 backbone, which alters the position of the C‐terminal part of the strand and the Cδ2–Cδ3 linker. A packing interaction between the strand and linker and Trp370, which is conserved in other isotypes, is also lost as a result of the conformational change. This unique feature in IgD‐Fc appears to be linked to the relative orientation of the Cδ2 and Cδ3 domains, which is discussed in more detail later.

The Cδ2 domain contains a high‐mannose oligosaccharide moiety covalently attached to Asn354 in the DE loop [25, 26] that was modeled in each chain as follows: Chains A and C, GlcNAc_2_Man_4_; Chain B, GlcNAc_2_Man_7_; and Chain D, GlcNAc_2_Man_6_. The high‐mannose oligosaccharide moiety observed in the crystal structure is consistent with the glycoanalysis of human serum and myeloma IgD [25, 26]. Due to the contact between the Cδ2 domains, a number of hydrogen bonds form between the carbohydrate residues attached to the different chains (Figure 2). This is a substantially greater interaction than is typically seen in other isotypes, whether high‐mannose or complex‐type oligosaccharides.

### 3.3 Cδ3 Domain Structure and Glycosylation

**FIGURE 4:**
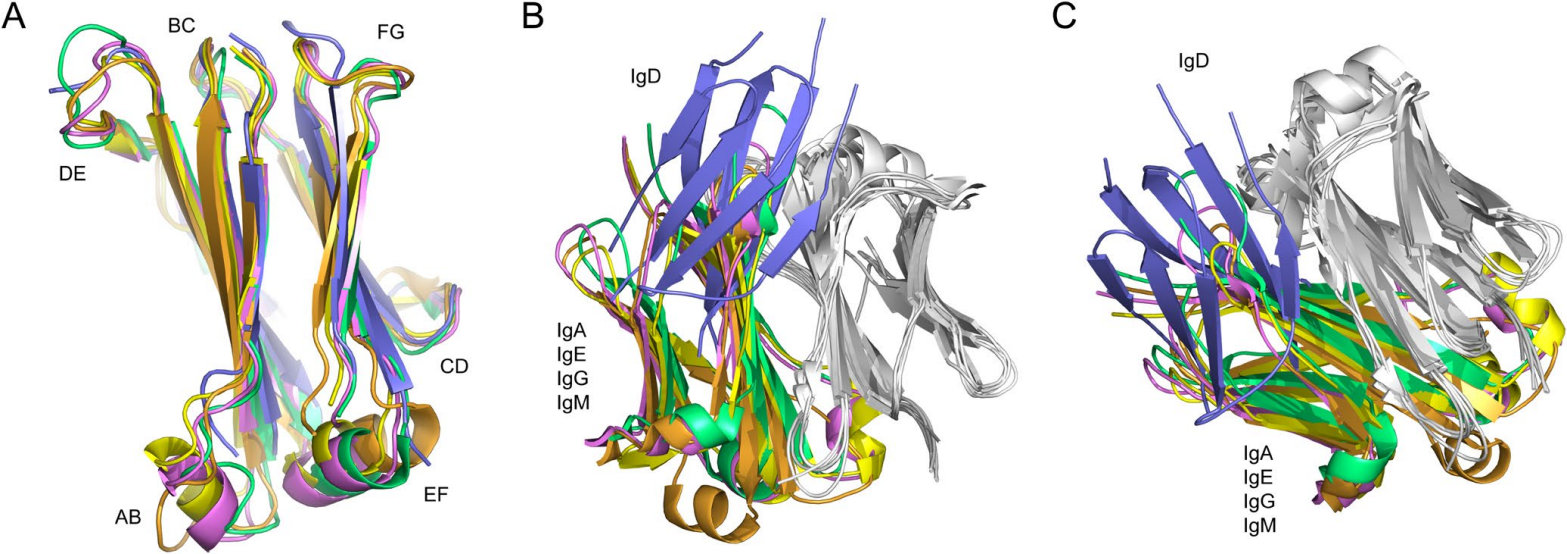
Overall structure of the Cδ3 domain. (A) With the exception of the flexible loop regions, the overall structure of the Cδ3 domain (Chain A, blue) is similar to that for Cα3 (IgA, green), Cε4 (IgE, orange), Cγ3 (IgG, yellow), and Cμ4 (IgM, purple). (B and C) The overall structure of the Cδ3 domain dimer is markedly different to those for Cα3, Cε4, Cγ3, and Cμ4. All structures were superposed on one domain from the dimer (light gray). The second domain is colored as follows: Cα3, green; Cε4, orange; Cγ3, yellow; Cμ4, purple; and Cδ3, blue. Panel (B) highlights the two‐fold symmetry of the Cα3, Cε4, Cγ3, and Cμ4 domain dimers, while panel (C) highlights the two‐fold symmetry of the Cδ3 domain dimer. The following structures were used to generate the figure: IgA, PDB: 1OW0 [21]; IgE, PDB: 5MOL [22]; IgG, PDB: 1L6X [23]; IgM, PDB: 8BPG [24]. In panels (B and C), the Cδ3 domain dimer is shown between Chains A and B.

Some regions of the Cδ3 domains (Pro401‐Leu501) displayed greater disorder than the Cδ2 domains and the AB, DE, and EF loops could not be modeled completely. The overall structure of the human Cδ3 domain is similar to that for the human Cα3, Cγ3, Cε4, and Cμ4 domains (Figure 4A). The conformation of the Cδ3 BC loop is like those in the other four isotypes while the Cδ3 CD loop is more akin to the Cγ3 CD loop. The parts of the Cδ2 domain AB and DE loops that could be modeled were different to those in the other isotypes. Furthermore, while the conformation of the FG loop is similar in IgA, IgE, IgG, and IgM (even though the FG loop in IgE contains a single‐residue insertion), in IgD the loop structure is different and forms a β‐hairpin.

The Cδ3 domain contains two N‐linked glycosylation sites at positions 445 and 496 to which complex‐type oligosaccharide moieties are attached [25, 26]. Although electron density was observed near Asn445 at low contour levels in both 2F_o_ − F_c_ and F_o_ − F_c_ maps, it could not be reasonably modeled as carbohydrate. However, a single N‐acetylglucosamine residue covalently attached to Asn496 was modeled in Chains A, B and D (Figure 1A).

### 3.4 The Cδ3 Domains Adopt a Unique Arrangement in the Cδ3 Domain Dimer

**FIGURE 5:**
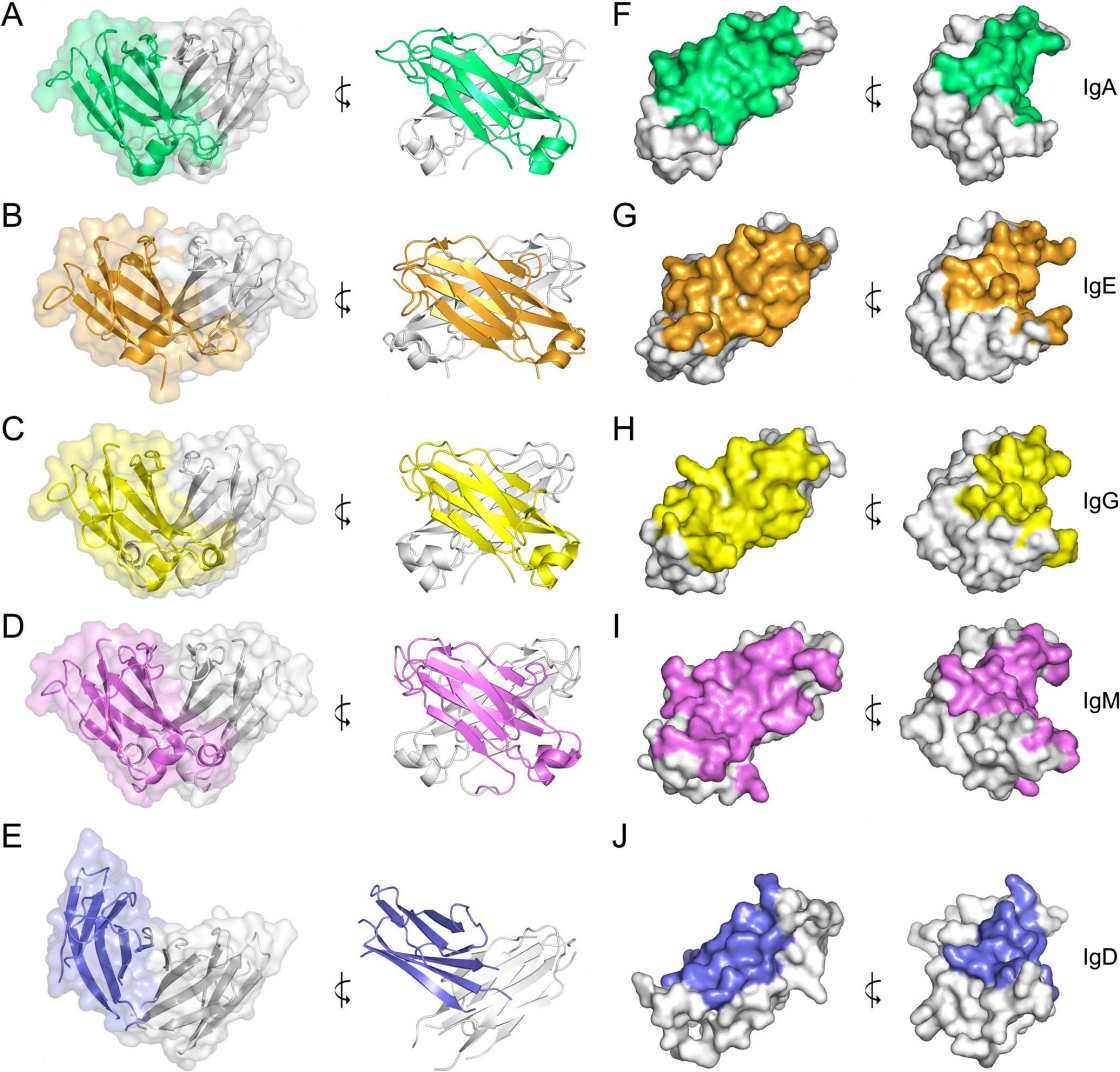
CH3 and CH4 domain dimer interfaces in human isotypes—overall structure. The domain dimers were superposed on one domain, which is colored light gray in panels (A–E). (A) Cα3 domain dimer in human IgA. The Cα3 domains are colored light gray and green. (B) Cε4 domain dimer in human IgE. The Cε4 domains are colored light gray and orange. (C) Cγ3 domain dimer in human IgG. The Cγ3 domains are colored light gray and yellow. (D) Cμ4 domain dimer in human IgM. The Cμ4 domains are colored light gray and purple. (E) Cδ3 domain dimer in human IgD. The Cδ3 domains are colored light gray and blue. The Cδ3 domain colored light gray is shown in the same orientation as the light gray domains in panels (A–D). The relative position of the domain colored blue is different to those in the other dimers. (F) Residues that form the Cα3 domain dimer interface in IgA (green) are indicated on a single Cα3 domain (light gray). (G) Residues that form the Cε4 domain dimer interface in IgE (orange) are indicated on a single Cε4 domain (light gray). (H) Residues that form the Cγ3 domain dimer interface in IgG (yellow) are indicated on a single Cγ3 domain (light gray). (I) Residues that form the Cμ4 domain dimer interface in IgM (purple) are indicated on a single Cμ4 domain (light gray). (J) Residues that form the Cδ3 domain dimer interface in IgD (blue) are indicated on a single Cδ3 domain (light gray). The following structures were used to generate the figure: IgA, PDB: 1OW0 [21]; IgE, PDB: 5MOL [22]; IgG, PDB: 1L6X [23]; IgM, PDB: 8BPG [24]. In panel (E), the Cδ3 domain dimer is shown between Chains A and B. In panel (J), the Cδ3 domain in Chain D was used to construct a model that incorporated residues that were disordered in Chain D, but modeled in Chains A–C. This model contained residues Pro401‐Asp413, Glu416‐Pro455, Arg459‐Pro474, and Gln478‐Leu501.

In human IgA‐, IgE‐, IgG‐, and IgM‐Fc, the CH3 (IgA and IgG) and CH4 (IgE and IgM) domains are arranged in a conserved manner and form a two‐fold symmetrical dimer [21, 22, 23, 24]. Although the Cδ3 domain dimer displays two‐fold symmetry, the relative orientation of the domains is markedly different to those in the other isotypes. When the CH3 and CH4 domain dimers are superposed on one of the domains, the second domain in IgD is rotated by ~35° relative to the second domain in the dimers (Figures 4B,C and 5A–E). The orientation of the domains within the Cδ3 domain dimer is thus unique among the human antibody isotypes.

The buried surface areas for the IgA, IgE, and IgG CH3/CH4 domain dimer interfaces range from ~1020 Å^2^ (IgA) to ~1200 Å^2^ (IgE). The buried surface area for the IgM Cμ4 interface is substantially lower at ~730 Å^2^ but that for the IgD Cδ3 interface is the lowest at ~640 Å^2^ (Figure 5F–J).

A number of strands and loops contribute to the CH3/CH4 domain dimer interface. In all isotypes, the interface includes β‐strands B, D, and E and the AB and DE loops. The interfaces in IgA, IgE, IgG, and IgM also include β‐strand A, the interfaces in IgE and IgG include β‐strand G, and the interfaces in IgE and IgM include the BC loop and the C‐terminal region, respectively. By contrast, the only other region in the Cδ3 domain that contributes to the dimer interface in IgD is the CD loop (which is also involved in the IgA, IgE, and IgM dimer interfaces).

**FIGURE 6:**
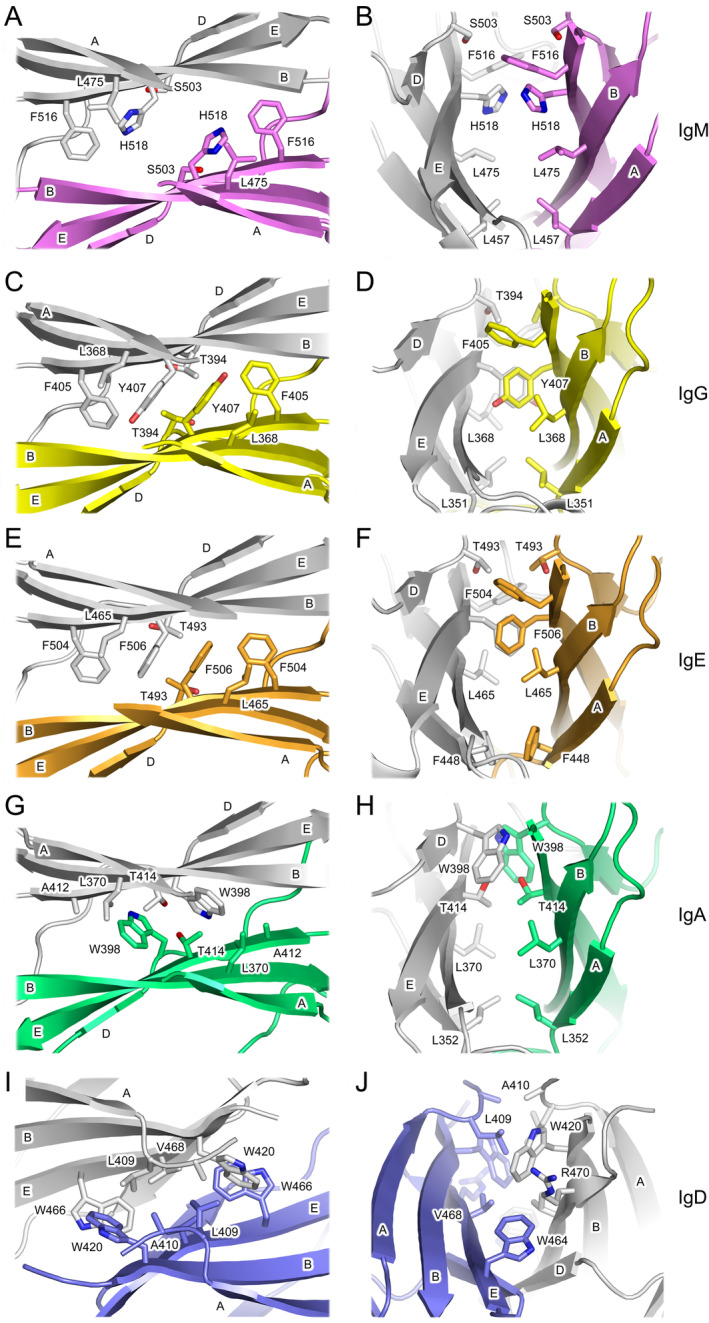
CH3 and CH4 domain dimer interfaces in human isotypes—detailed interactions. (A and B) Interface between the Cμ4 domains in IgM. The Cμ4 domains are colored light gray and purple. (C and D) Interface between the Cγ3 domains in IgG. The Cγ3 domains are colored light gray and yellow. (E and F) Interface between the Cε4 domains in IgE. The Cε4 domains are colored light gray and orange. (G and H) Interface between the Cα3 domains in IgA. The Cα3 domains are colored light gray and green. (I and J) Interface between the Cδ3 domains in IgD. The Cδ3 domains are colored light gray and blue. The following structures were used to generate the figure: IgA, PDB: 1OW0 [21]; IgE, PDB: 5MOL [22]; IgG, PDB: 1L6X [23]; IgM, PDB: 8BPG [24]. In panels (I and J), the Cδ3 domain dimer is shown between Chains A and B. β‐strands are labeled where appropriate.

The dimer interfaces in IgA, IgE, IgG, and IgM share some similar features, which will be described before the differences that are found in IgD are discussed. In IgM, IgG, and IgE, an aromatic residue on β‐strand E (His518 in IgM, Tyr407 in IgG, and Phe506 in IgE) (Figure 3) forms a stacking interaction at the symmetry axis of the dimer (Figure 6A–F), although Tyr407 in IgG and Phe506 in IgE form more substantial packing interactions than His518 in IgM. This aromatic residue is flanked by a small polar residue on β‐strand D (Ser in IgM, Thr in IgG and IgE) and invariant leucine and phenylalanine residues on β‐strands B and E, respectively (Figures 3 and 6A–F). On the other hand, in IgA (Figures 3 and 6G,H), the aromatic residue found on β‐strand E in IgM, IgG, and IgE is substituted for threonine, but the small polar residue on β‐strand D is substituted for tryptophan, which preserves the stacking interaction of aromatic residues at the interface. The phenylalanine residue on β‐strand E that is conserved in IgM, IgG, and IgE is substituted for alanine in IgA, but the leucine residue on β‐strand B is unchanged.

In IgD, the nature of the interfacing residues at the CH3 domain dimer interface is strikingly different to those in other isotypes (Figure 6I,J). One of the most notable differences at the Cδ3/Cδ3 interface is the absence of a stacking interaction between aromatic residues at the symmetry axis of the dimer. Residues with smaller side chains, including Ala410 (AB loop), Gly448 (β‐strand D), and Val468 (β‐strand E) are found here instead. Val468 is flanked by Trp466 that forms a T‐shaped stacking interaction with Trp420 (β‐strand B) in the second Cδ3 domain. Trp420 in turn packs against Leu409 in the first domain. Trp466 is flanked by Trp464 in the same domain, which contacts Arg470 (β‐strand E) in the second domain; this interaction lies at the edge of the Cδ3 domain dimer interface. Another arginine residue, Arg453 (DE loop), is also located at the edge of the dimer interface but, unlike Arg470, Arg453 adopts a substantially different conformation in each of the four chains and does not form a consistent interaction with nearby residues. This region is also a site of structural diversity at the interfaces in other isotypes.

Among the human isotypes, IgD is therefore unique not only in the overall arrangement of the Cδ3 domain dimer, but in the nature of the interactions that form at the dimer interface.

### 3.5 The Cδ2 and Cδ3 Domains Form a Larger Interface Compared With Other Isotypes

**FIGURE 7:**
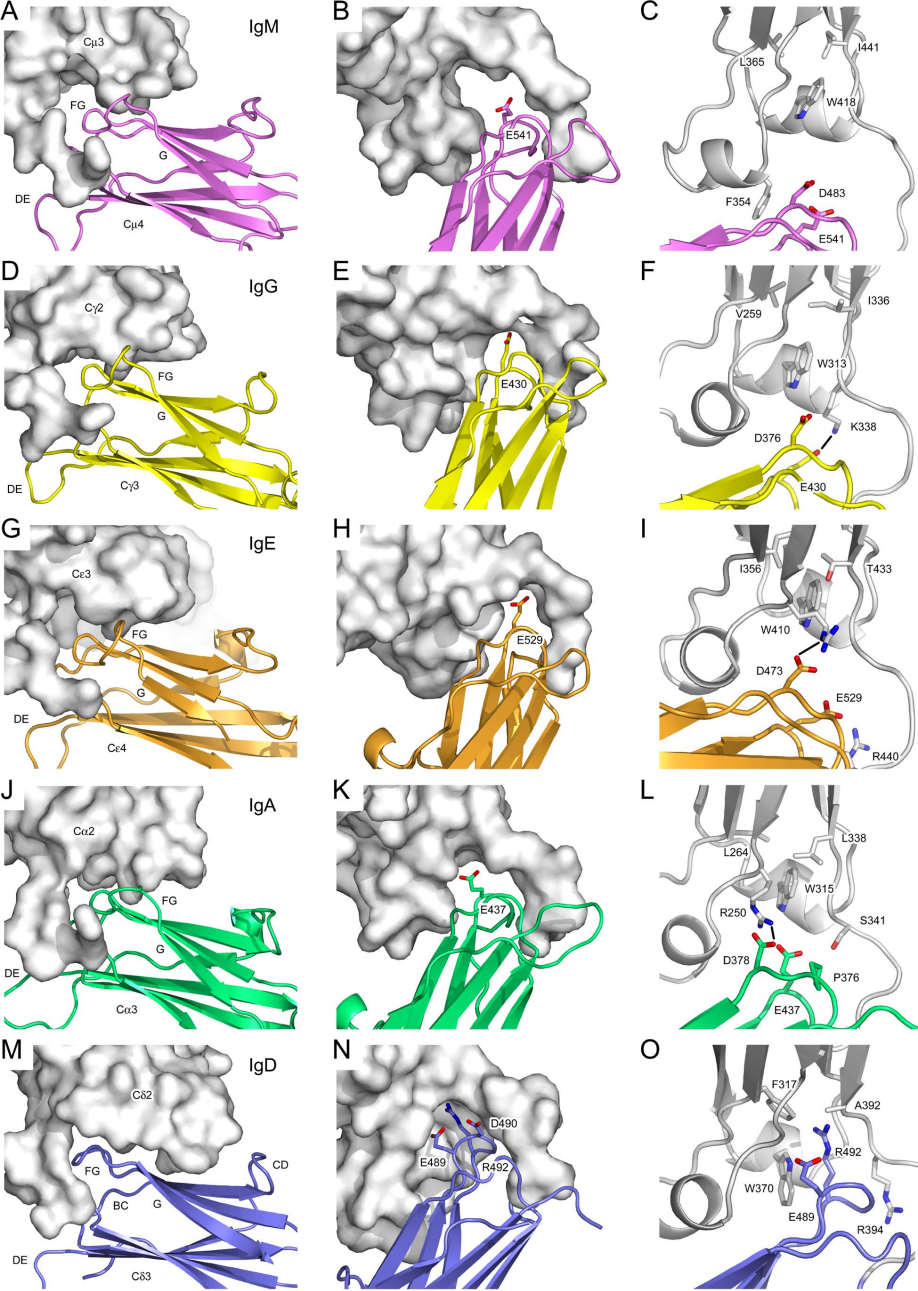
CH2/CH3 and CH3/CH4 domain dimer interfaces in human isotypes. (A–C) Interface between the Cμ3 domain and Cμ3–Cμ4 linker (light gray) and Cμ4 domain (purple) in IgM. D483 and E541 are located in the Cμ4 domain BC and FG loops, respectively. (D–F) Interface between the Cγ2 domain and Cγ2–Cγ3 linker (light gray) and Cγ3 domain (yellow) in IgG. D376 and E430 are located in the Cγ3 domain BC and FG loops, respectively. (G–I) Interface between the Cε3 and Cε3–Cε4 linker (light gray) and Cε4 domain (orange) in IgE. D473 and E529 are located in the Cε4 domain BC and FG loops, respectively. (J–L) Interface between the Cα2 and Cα2–Cα3 linker (light gray) and Cα3 domain (green) in IgA. P376 and D378 are located in the Cα3 domain BC loop and E437 is located in the FG loop. (M–O) Interface between the Cδ2 domain and Cδ2–Cδ3 linker (light gray) and Cδ3 domain (blue) in IgD. E489, D490 and R492 are located in the Cδ3 domain FG loop. The interface in Chain A is shown. The following structures were used to generate the figure: IgA, PDB: 1OW0 [21]; IgE, PDB: 5G64 [59]; IgG, PDB: 1L6X [23]; IgM, PDB: 8BPG [24]. The structures were superposed on the IgD Cδ2 domain. Any disordered residues were modeled in Coot [15] using standard rotamers. In panels (F, I, and L), salt bridges are depicted by black lines.

In all human antibody isotypes, the CH2 (IgA, IgD, and IgG) and CH3 (IgE and IgM) domains contact the CH3 (IgA, IgD and IgG) and CH4 (IgE and IgM) domains, respectively (Figure 7). Together with their respective linkers, the buried surface areas for these interfaces in IgA (Figure 7J), IgE (Figure 7G), IgG (Figure 7D), and IgM (Figure 7A) range from 600 Å^2^ (IgM) to 715 Å^2^ (IgG). In IgD (Figure 7M), the interface between the Cδ2 domain/Cδ2–Cδ3 domain linker and the Cδ3 domain is significantly larger at 922 Å^2^. If the domains alone are considered, and the linker excluded, then the buried surface areas for the interfaces in IgA, IgE, IgG, and IgM range from 254 Å^2^ (IgM) to 352 Å^2^ (IgE), while that for IgD is 674 Å^2^.

At the interfaces in IgA (Figure 7J–L), IgE (Figure 7G–I), and IgG (Figure 7D–F), the Cα2, Cε3, and Cγ2 domains, and the inter‐domain linker, contact the respective Cα3, Cε4, and Cγ3 domain BC and FG loops, and salt bridges are found at each interface. Comparable loop interactions are found in IgM (Figure 7A–C), but the contact area with each Cμ4 domain loop is significantly lower compared with the other isotypes, and there are no salt bridges at the interface.

Due to the unique arrangement of the Cδ2 and Cδ3 domains in IgD, the contact area with the Cδ3 domain BC loop is smaller (67 Å^2^ in IgD compared with a range of 148 Å^2^ (IgM) to 237 Å^2^ (IgG)). However, there is a comparatively larger contact area with the Cδ3 CD loop (58 Å^2^) and Cδ3 β‐strand G (25 Å^2^).

**FIGURE 8:**
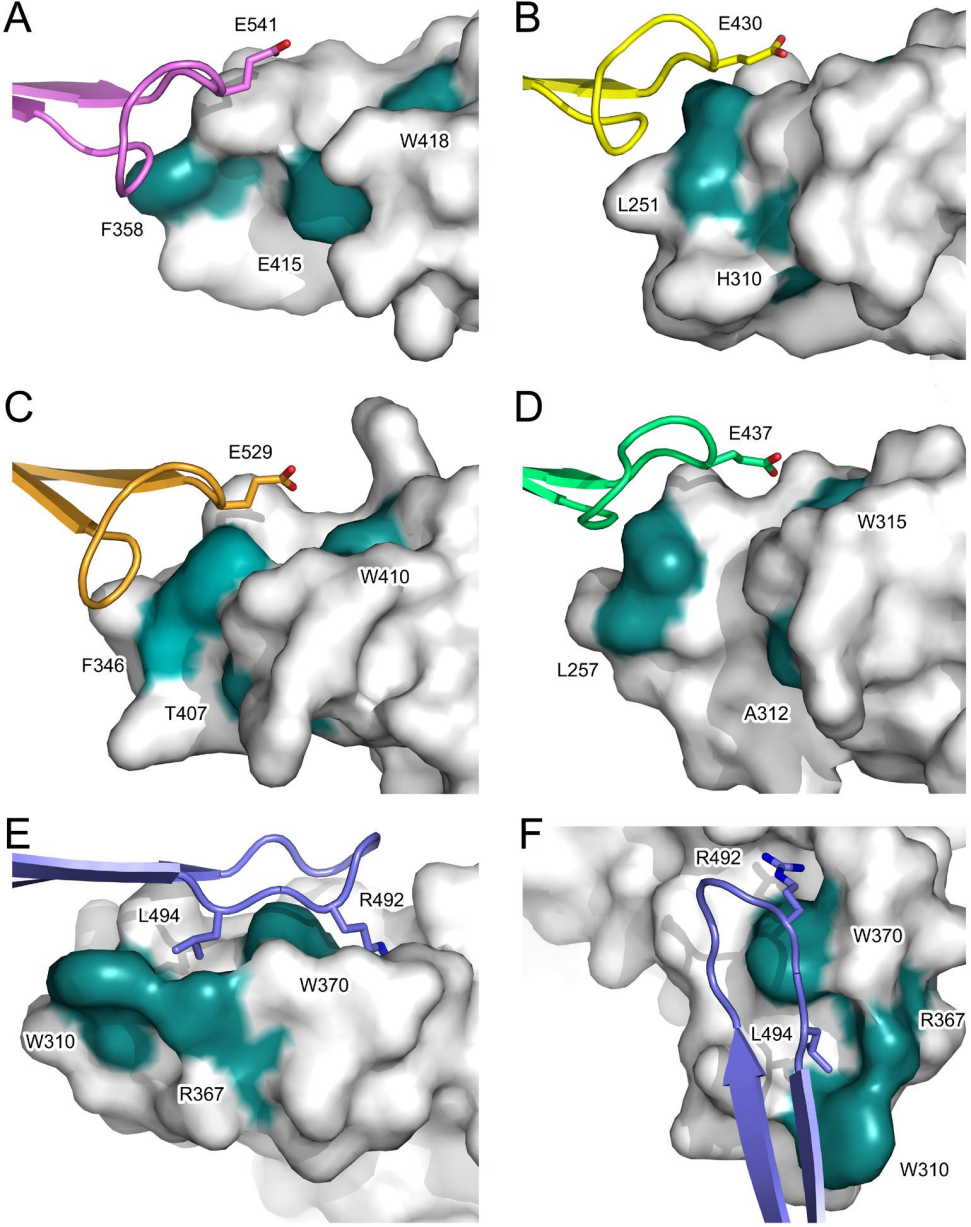
CH2/CH3 domain surface. In each figure, the position of a conserved tryptophan (Trp370 in IgD) and residues that are structurally equivalent to W310 and R367 in the IgD Cδ2 domain are colored in teal and labeled. The surface of the Cδ2 domain forms two pockets that accommodate residues from the Cδ3 domain FG loop, while the surfaces of the CH2/CH3 domains in the other isotypes lack equivalent pockets. (A) Interface between the Cμ4 domain FG loop (purple) and the Cμ3 domain (light gray). (B) Interface between the Cγ3 domain FG loop (yellow) and the Cγ2 domain (light gray). (C) Interface between the Cε4 domain FG loop (orange) and the Cε3 domain (light gray). (D) Interface between the Cα3 domain FG loop (green) and the Cα2 domain (light gray). (E) Interface between the Cδ3 domain FG loop (blue) and Cδ2 domain (light gray). (F) Interface between the Cδ3 domain FG loop (blue) and Cδ2 domain (light gray), showing a view of the two pockets from a different orientation to that shown in (E). The following structures were used to generate the figure: IgA, PDB: 1OW0 [21]; IgE, PDB: 5G64 [59]; IgG, PDB: 1L6X [23]; IgM, PDB: 8BPG [24]. Any disordered residues were modeled in Coot [15] using standard rotamers.

Strikingly, the interface with the Cδ3 domain FG loop is more than double that in other isotypes (519 Å^2^ in IgD compared with a range of 195 Å^2^ (IgM) to 251 Å^2^ (IgA)) (Figure 7). The large interface between the Cδ2 domain and Cδ3 FG loop is partly due to the structural differences found in Cδ2 compared with other human isotypes, as discussed earlier. Trp370 (Cδ2 domain EF loop) adopts a different rotamer in IgD compared with other isotypes, creating two pockets on each face of the aromatic ring that are almost reminiscent of a two‐pronged pocket (Figure S4 and Figures 7C,F,I,L,O and 8). The first pocket is bordered by Phe317 (Cδ2 β‐strand B), Trp370, Asn371, Gly373, Thr374 (Cδ2 EF loop), and Ala392 (Cδ2 β‐strand G). The side chain of Arg492 from the Cδ3 domain FG loop is partially buried in this first pocket (Figures 7M and 8E,F). By contrast, Arg492 is substituted for small, hydrophobic side chains in the other isotypes (Figure 3) and the pocket is also absent (Figures 7B,E,H,K and 8A–D).

The second pocket is also bordered by Trp370 and Asn371 (Cδ2 EF loop), and additionally by Val306, Leu309, Trp310 (Cδ2 AB loop), and Arg367 (Cδ2 EF loop), and accommodates Leu494 from the Cδ3 domain FG loop (Figure 8E,F). Key to the formation of this pocket in IgD are Trp310 and Arg367. In the other human isotypes, Trp310 is substituted for smaller hydrophobic and aromatic residues while Arg367 is substituted for a variety of different residue types (Figure 3), which are all too small to enclose the edge of the pocket (Figure 8A–D).

Interestingly, these pockets in the Cδ2 domain, at the Cδ2/Cδ3 domain interface, differ from a pocket at the Cγ2/Cγ3 interface in IgG (Figure S5) [27]. In IgG, a pocket is found on the Cγ3 domain that accommodates a leucine residue from the Cγ2 domain, creating a “ball‐and‐socket” joint, which acts as a pivot around which the Cγ2 domain moves [27].

In IgD, the Cδ2 and Cδ3 domains clearly form more extensive, intimate contacts than equivalent domains in the other isotypes. These contacts could restrict the ability of the Cδ2 domains to adopt different positions relative to the Cδ3 domains and the potential for conformational flexibility in IgD‐Fc, as seen in IgG‐Fc and more so in IgE‐Fc.

### 3.6 Sequence Conservation in Mammalian IgD

Given that the orientation of domains within the Cδ3 dimer is unique among the human isotypes, we wanted to determine whether the most important interfacing residues in human IgD were conserved in other mammalian species (Figure S6). We searched GenBank using the sequence for human IgD‐Fc and included mammalian sequences that had been clearly annotated as IgD.

Of the residues that are found at the interface of the human Cδ3 domain dimer, Ala410 and Val468 are conserved or substituted in a conservative manner (Figure S6). Ala410 is found in nonhuman primates and, of the sequences that were compared, is substituted for threonine in other species, with a single exception. Likewise, Val468 is conserved in most species, but substituted for leucine or isoleucine in others. By contrast, Gly448 is substituted for alanine, arginine, asparagine, glutamine, and, intriguingly, tryptophan (Figure S6). Trp464, located at the edge of the interface, is conserved mainly in nonhuman primates and, with one exception, substituted for histidine or glutamine in other species. Arg470, also located at the edge of the interface, is conserved in many species, and substituted mostly for histidine in others.

A key interaction at the Cδ3 domain dimer interface is a T‐shaped stacking interaction between Trp420 in one domain and Trp466 in the other. These residues are invariant in all the mammalian species that were included in the sequence analysis (Figure S6).

Another unique feature in human IgD is the packing of Arg492 and Leu494 from the Cδ3 domain FG loop into two pockets on the surface of the Cδ2 domain. Arg492 is invariant in IgD‐Fc regions that contain both Cδ2 and Cδ3 domains (Figure S6). Arg492 is substituted for lysine and glutamine in mouse and rat IgD‐Fc, respectively, but IgD in these species lack a Cδ2 domain [28, 29]. Likewise, Leu494 is highly conserved, substituted for valine in only two species, but is replaced by lysine in mouse and rat. Of the most important residues that form the pockets on the Cδ2 domain surface, Trp370, which adopts a different conformation in IgD compared with other human isotypes, is invariant. Phe317, which imposes steric constraints on the conformation of Trp370, is also invariant. Trp310, which forms part of the border at one edge of the Leu494 pocket, is invariant, while Arg367, which forms the other part of the border, is highly conserved, and substituted for valine or isoleucine in only three species (Figure S6).

Thus, residues that contribute to the unique features human IgD‐Fc are invariant or highly conserved in a variety of mammalian species.

### 3.7 Human IgD‐Fc Unfolds Thermally in a Single Transition

**FIGURE 9:**
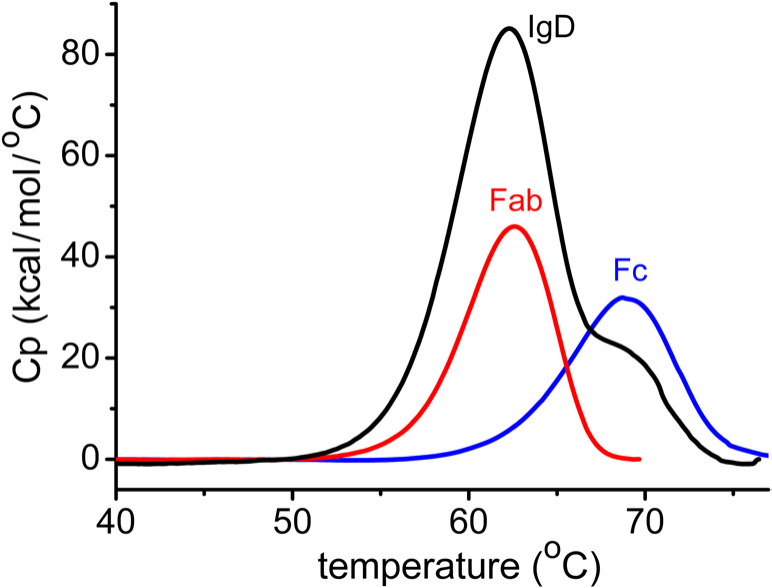
Thermal stability of IgD. Baseline‐corrected differential scanning calorimetry data for an anti‐Phl p 7 IgD Fab containing a λ chain (23.9 μM, red), IgD‐Fc (43.9 μM, blue), and intact anti‐Phl p 7 IgD (15.1 μM, black). The IgD Fab and IgD‐Fc unfold in a single transition with melting temperatures of 62.4°C and 68.6°C, respectively. Intact IgD unfolds in two transitions with melting temperatures of 62.1°C and 69.4°C that correspond to the Fab fragments and Fc region, respectively. The experiments were performed in PBS pH 7.4 and 0.1% (w/v) sodium azide.

We investigated the thermal stability of IgD, IgD‐Fc, and an IgD Fab fragment using DSC. The thermograms for the thermal denaturation of IgD‐Fc at concentrations of 17.5 and 43.9 μM each revealed a single transition with melting temperatures of 68.7°C and 68.6°C, respectively (Figure 9). The thermal stability of IgD‐Fc was also studied in the context of an intact human IgD antibody. This particular antibody is specific for the Phl p 7 grass pollen allergen and has been described previously [5, 6]. Firstly, the thermal stability of the IgD Fab fragment from this antibody, which contains a λ light chain, was determined. The IgD Fab unfolded in a single transition with a melting temperature of 62.4°C, a value that is consistent with IgG Fab fragments that contain λ chains [30]. The thermal unfolding of the intact IgD antibody revealed two transitions, with melting temperatures of 62.1°C and 69.4°C, corresponding to the Fab fragments and Fc region, respectively. Thus, the thermal stability of the IgD‐Fc region is not significantly altered in the intact antibody.

## 4 Discussion

The overall structure of IgD‐Fc is markedly different to the Fc regions of antibodies of all the other isotypes. Indeed, among the human isotypes, the orientation of the Cδ3 domains within their dimer, and the nature and arrangement of the interfacing residues, is unique. Furthermore, as part of the extensive interface between the Cδ2 and Cδ3 domains, the largest among the human isotypes, the Cδ2 domain uniquely forms two pockets that accommodate residues in the Cδ3 domain FG loop.

### 4.1 Is Human IgD‐Fc Conformationally Flexible?

The Fc regions of antibodies are conformationally flexible [31, 32, 33]. In IgE in particular, the Cε3 domains, which are equivalent to the Cδ2 domains in IgD, adopt “open” and “closed” conformations that regulate receptor binding in an allosteric, mutually exclusive manner [34, 35]. However, even in a closed conformation, there are no protein–protein contacts between the Cε3 domains. Therefore, the Cδ2 domains in IgD, with the substantial interface between their hinge‐proximal regions, could be described as adopting a fully closed conformation.

It is currently unknown if IgD‐Fc is as conformationally flexible as the Fc regions of other isotypes. The Cδ2 and Cδ3 domains form an extensive interface and, in contrast to other isotypes, the Cδ2 domain surface contains two pockets that accommodate residues from the Cδ3 domain; the interface area is the largest among the human isotypes. Together with the interface between the hinge‐proximal regions of the Cδ2 domain, and the contacts between the oligosaccharide moieties covalently attached to each Cδ2 domain that in effect “bridge” the two domains, this extensive Cδ2/Cδ3 interface could restrict the ability of the Cδ2 and Cδ3 domains to adopt a range of different positions relative to one another. At 64 residues in length, IgD has the longest hinge region of the human isotypes, yet the hinge contains only a single disulfide bond. By contrast, IgG3 has a hinge that is 62 residues in length, with 11 disulfide bonds. The extensive interfaces in IgD‐Fc involving the Cδ2 domains could be a mechanism to stabilize the homodimer, perhaps at the expense of flexibility.

If there is conformational flexibility in IgD‐Fc, perhaps required for IgD function, it could be that this is mediated primarily through the Cδ3 domains, with their relatively small dimer interface area, and not Cδ2. (This would be in contrast to IgG and IgE, in which the Cγ2 and Cε3 domains move relative to the unchanged Cγ3 and Cε4 domain dimers). However, in the absence of any experimental evidence, this is purely speculative. The functional properties of IgD in humans and other mammals is still poorly understood, but it is noteworthy that mouse and rat IgD‐Fc are composed of only Cδ3 domains.

### 4.2 Thermal Stability of Human IgD‐Fc

Human antibody Fc regions behave differently upon thermally‐induced unfolding. Human IgE‐Fc, with three constant domains (Cε2, Cε3, and Cε4), unfolds in two steps with melting temperatures of ~55°C and ~ 64°C, while the Fcε3‐4 subfragment unfolds in a single step with a melting temperature of ~52°C [22]. These data have been interpreted as the Fcε3‐4 subfragment unfolding at the lower temperature, probably in a cooperative manner, with the Cε2 domain pair unfolding later [22]. Human IgG1‐Fc, with only two domains in its Fc region, also unfolds in two steps with melting temperatures of ~65°C–71°C and ~ 82°C, which corresponds to unfolding of the Cγ2 and Cγ3 domains, respectively [36, 37, 38]. By contrast, human IgG4‐Fc unfolds in a single step, with a melting temperature of ~65°C [36].

Thermally‐induced unfolding of IgD‐Fc is more similar to that of IgG4‐Fc and the IgE Fcε3‐4 subfragment in that it unfolds in a single step. However, the melting temperature of IgD‐Fc (~68.6°C) is higher than for IgG4‐Fc and IgE Fcε3‐4. In the case of IgG4‐Fc, it was not possible to determine whether the Cγ2 and Cγ3 domains unfolded with identical melting temperatures, or a single cooperative unit [36]. Unfolding of Fcε3‐4 was suggested to be cooperative, and the flexible, molten‐globule‐like Cε3 domains the most thermally labile part of IgE‐Fc. By contrast, the interface between the Cδ2 domains, the contact between the oligosaccharide moieties from different Cδ2 domains, and the relatively low interface area between the Cδ3 domains suggest that the Cδ3 domains might be the more labile part of IgD‐Fc. However, it remains possible that the Cδ2 and Cδ3 domains unfold with similar melting temperatures.

The melting temperature of IgD‐Fc is not substantially altered in the intact antibody, which suggests that the Fabs and hinge region do not contribute to the thermal stability of the Fc region. The DSC data are consistent with the solution structure of IgD [4], which revealed a predominantly T‐shaped molecule with a semi‐extended hinge in which there was no contact between the Fabs and Fc region.

### 4.3 Implications for Human IgD Function

Like the other human antibody isotypes, human IgD exists in both membrane‐bound (as part of the B‐cell receptor) and secreted forms. However, unlike the other isotypes, the function of secreted IgD is still relatively poorly understood.

The recent cryo‐electron microscopy structures of the human IgG and IgM B‐cell receptors [39, 40] reveal different orientations of the IgG‐ and IgM‐Fc regions relative to the Igα/Igβ heterodimer. When the human IgD‐Fc structure was compared with that of the human IgM B‐cell receptor, steric clashes were observed between one of the Cδ2 domains and the Igα chain as a result of the unique arrangement of the Cδ2 and Cδ3 domains relative to one another in IgD‐Fc (Figure S7). A similar comparison with the IgG B‐cell receptor revealed potential clashes between the Cδ3 domain EF loop and the Igβ chain, but this loop is partially disordered in the IgD‐Fc crystal structure. Although the comparison with the IgG B‐cell receptor structure revealed fewer potential clashes than with the IgM B‐cell receptor, the linker between the Fc region and transmembrane region is longer in IgD than in IgG. The overall structure of the IgD B‐cell receptor could therefore differ substantially from those for IgM and IgG.

The human IgA, IgE, IgG, and IgM antibody isotypes bind a variety of receptors at different sites on their Fc regions and the molecular basis for many of these interactions is well‐understood. By contrast, the cellular receptors for IgD are less well‐characterized [2], but recent evidence suggests that secreted IgD can bind CD44 in a galectin‐9‐mediated interaction [3]. The IgD hinge region is heavily glycosylated and there are three glycosylation sites in the Fc region, one in the Cδ2 domain (Asn354) and two in the Cδ3 domain (Asn445 and Asn496). The glycosylation site at Asn445 is conserved in nonhuman primates while the site at Asn496 is more widely conserved. The site at which galectin‐9 binds IgD to mediate the interaction with CD44 is currently unknown.

A detailed analysis of the human IgD‐Fc structure with those of the other human isotypes in complex with their receptors is beyond the scope of this paper. However, an initial comparison suggests that the interaction between IgD and its receptor(s) could be very different. For example, IgE and IgG engage the structurally similar FcεRI and Fcγ receptors, respectively, through the parts of the Cε3 and Cγ2 domains that are distal to the Cε4 and Cγ3 domains, and binding to these receptors involves an “open” domain conformation [41, 42, 43, 44]. In human IgD, the Cδ2 domains are too close together to engage a receptor in a similar manner (Figure S8).

Exposed regions of inter‐domain interfaces in the Fc region are also utilized as receptor binding sites (Figure S8). For example, IgA binds FcαRI at the Cα2/Cα3 domain interface [21], IgE binds CD23 at the Cε3/Cε4 domain interface, [35] and IgG binds FcRn and TRIM21 at the Cγ2/Cγ3 domain interface [45, 46]. In IgA and IgG, the same interface serves additionally as a binding site for microbial proteins [47, 48, 49, 50, 51]. In principle, the Cδ2/Cδ3 interface could act as a receptor binding site, but the interface in IgD would differ from those in the IgA‐ and IgG‐receptor complexes due to the relative arrangement of the Cδ2 and Cδ3 domains. However, it is also noteworthy that not all mammalian IgD‐Fc regions contain a Cδ2 domain, and any receptor that engaged only the Cδ3 domain, which is not unprecedented given the interaction between FcμR and the Cμ4 domain [24], would need to do so in a manner that avoided steric clashes with the oligosaccharide moiety.

### 4.4 Sequence Conservation in Mammalian IgD

IgD‐Fc displays extraordinary structural diversity between species and even among mammals. Although many mammalian IgD‐Fc regions comprise Cδ2 and Cδ3 domains, mouse and rat IgD‐Fc contain only the Cδ3 domain [28, 29, 52, 53, 54, 55] (Figure S6), while platypus IgD‐Fc comprises nine constant domains and is more similar to IgD in reptiles and fish [56].

Of the mammalian IgD sequences that were compared, many of the determinants that are associated with the unique structural properties of human IgD‐Fc were invariant, highly conserved, or substituted in a conservative manner, with the implication that the features observed in human IgD‐Fc could also be found in other species. However, in the platypus, IgD‐Fc domains that are homologous to human Cδ2 and Cδ3 [56], key residues that are involved at inter‐domain interfaces, particularly at the Cδ2/Cδ3 interface, do not appear to be as widely conserved as in other mammals.

A residue in human IgD‐Fc that displays great diversity in other species is Gly448, which is substituted for alanine, arginine, asparagine, glutamine, and even tryptophan (Figure S6). This residue is located on β‐strand D, which is an edge strand at the Cδ3/Cδ3 domain interface, closest to the internal part of the molecule. Modeling suggests that sequence variation at position 448 could cause substantial local structural changes at the Cδ3/Cδ3 interface, and even affect the relative orientation of the domains within the dimer. However, given how different human IgD‐Fc is to the other human isotypes, and the diversity in IgD‐Fc found in animals such as bony fish [57], structural variation at the mammalian Cδ3/Cδ3 interface is perhaps to be expected.

## 5 Conclusion

In summary, the 3.0 Å resolution crystal structure of human IgD‐Fc reveals a uniquely different disposition of its four domains, compared with the homologous region of all other antibodies whose structures are known. In particular, there are stronger interactions between the Cδ2 domains and with Cδ3, and weaker interactions between the Cδ3 domains, compared with the homologous domains of antibodies of other isotypes. These differences suggest that if there are conformational changes in IgD‐Fc associated with its function, they may involve structural changes in the Cδ3 domains rather than in Cδ2, in contrast to the conformational changes seen in the Fc regions of other human isotypes, notably IgG and IgE.

## Author Contributions

**Anna M. Davies:** conceptualization, formal analysis, investigation, resources, data curation, writing – original draft, writing – review and editing, visualization. **Tam T. T. Bui:** investigation, writing – review and editing, formal analysis. **Raúl Pacheco‐Gómez:** writing ‐ review and editing, formal analysis, resources. **Susan K. Vester:** resources, writing – review and editing. **Andrew J. Beavil:** resources, writing – review and editing, funding acquisition. **Hannah J. Gould:** funding acquisition, writing – review and editing. **Brian J. Sutton:** writing – review and editing, funding acquisition. **James M. McDonnell:** conceptualization, data curation, writing – original draft, writing – review and editing, visualization, supervision, project administration, funding acquisition.

## Conflicts of Interest

Raúl Pacheco‐Gómez, Principal Field Application Specialist working for Malvern Panalytical Ltd. has collaborated with the authors of the scientific article “The crystal structure of human IgD‐Fc reveals unexpected differences with other antibody isotypes.” The other authors declare no conflicts of interest.

### Peer Review

The peer review history for this article is available at https://www.webofscience.com/api/gateway/wos/peer‐review/10.1002/prot.26771.

## Supporting information


Data S1.

## Data Availability

Coordinates and structure factors have been deposited in the Protein Data Bank with PDB ID 9FMB.
