# Supplementary material for: The Crystal Structure of Human IgD‐Fc Reveals Unexpected Differences With Other Antibody Isotypes
Source: Proteins. 2024 Nov 25;93(4):786–800. doi: 10.1002/prot.26771 (PMC11878202; doi:10.1002/prot.26771)
Supplement: Supplementary file 1 — Data S1. [file PROT-93-786-s001.docx]

**Supporting Information**

**The crystal structure of human IgD-Fc reveals unexpected differences with other antibody isotypes**

Anna M. Davies^1^, Tam T. T. Bui^2^, Raúl Pacheco-Gómez^3^, Susan K. Vester^1^, Andrew J. Beavil^1^, Hannah J. Gould^1^, Brian J. Sutton^1^ and James M. McDonnell^1^

**Affiliations**

^1^ King’s College London, Randall Centre for Cell and Molecular Biophysics, New Hunt’s House, London, SE1 1UL, United Kingdom. ^2^ King’s College London, Centre for Biomolecular Spectroscopy, London, SE1 1UL, United Kingdom. ^3^ Malvern Panalytical Ltd, Enigma Business Park, Grovewood Road, Malvern, WR14 1XZ, United Kingdom.

**Corresponding author**

James M. McDonnell

King’s College London

Randall Centre for Cell and Molecular Biophysics

New Hunt’s House

Guy’s Campus

London

SE1 1UL

United Kingdom

Tel: + 44 (0) 20 7848 6970

E-mail: james.mcdonnell@kcl.ac.uk

**
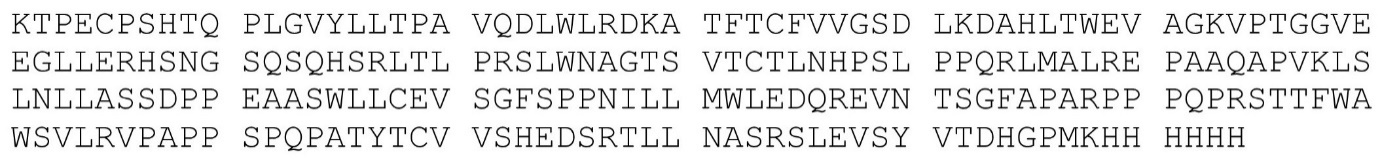
**

**Figure S1.** Human IgD-Fc sequence. The sequence of the human IgD-Fc construct used in this study.


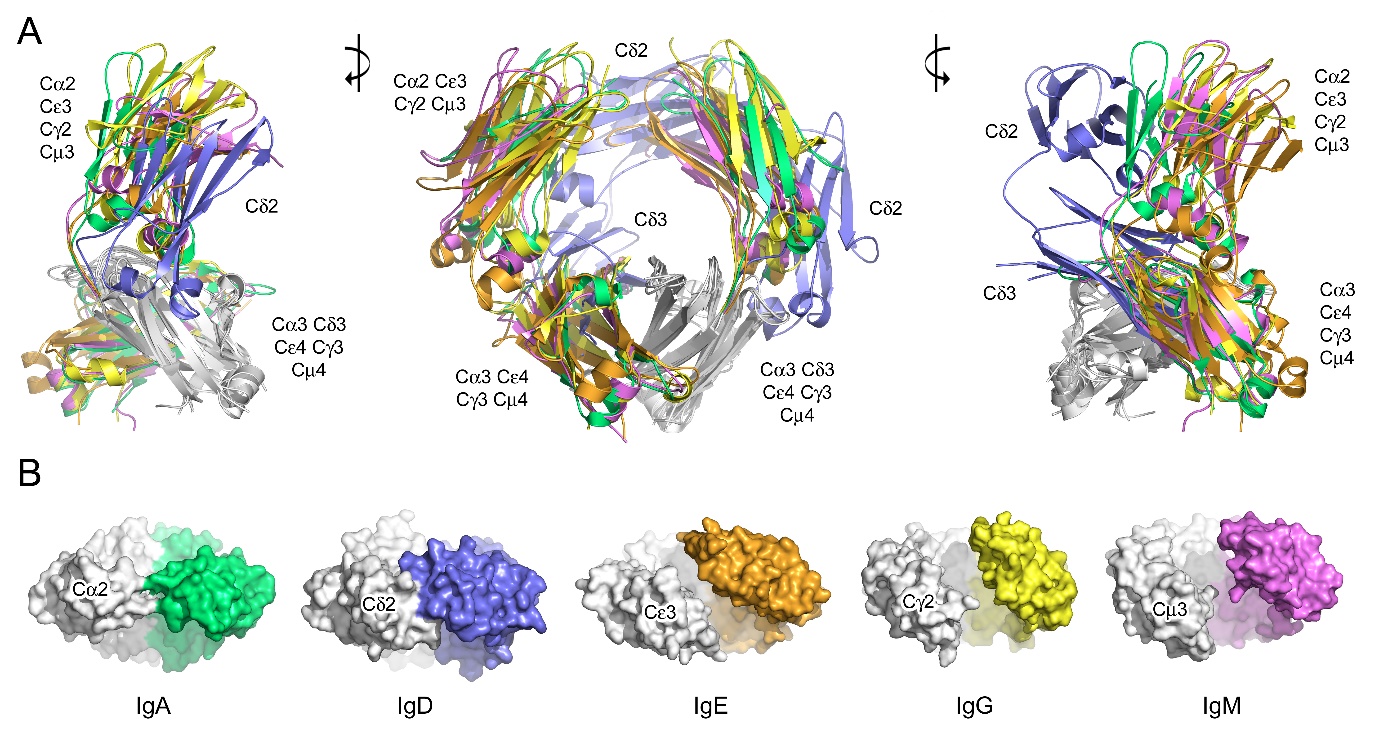


**Figure S2.** Overall architecture of human Fc regions. (A) Superposition of antibody Fc structures (IgA-Fc, green; IgD-Fc, blue; IgE-Fcε3-4, orange; IgG-Fc, yellow; IgM-Fcμ3-4, purple) reveals substantial differences in the disposition of the domains in IgD-Fc. The superposed C_H_3 and C_H_4 domains are coloured in light grey. The following structures were used to generate the figure: IgA, PDB: 1OW0^1^; IgE, PDB: 5MOL^2^; IgG, PDB: 3AVE^3^; IgM, PDB: 6KXS.^4^ (B) A view of antibody Fc structures that shows the positions of the C_H_2 (Cα2, Cδ2 and Cγ2) and C_H_3 domains (Cε3 and Cμ3) domains relative to one another. The IgE-Fcε3-4 structure is for the closed conformation involved in CD23 receptor binding. Of all the human isotypes, the interface between the Cδ2 domains buries the largest surface area. For each structure, one chain is coloured light grey and the other chain is coloured as follows: IgA, green; IgD, blue; IgE, orange; IgG, yellow and IgM, purple. The following structures were used to generate the figure: IgA, PDB: 1OW0^1^; IgE, PDB: 4EZM^5^; IgG, PDB: 3AVE^3^; IgM, PDB: 6KXS.^4^


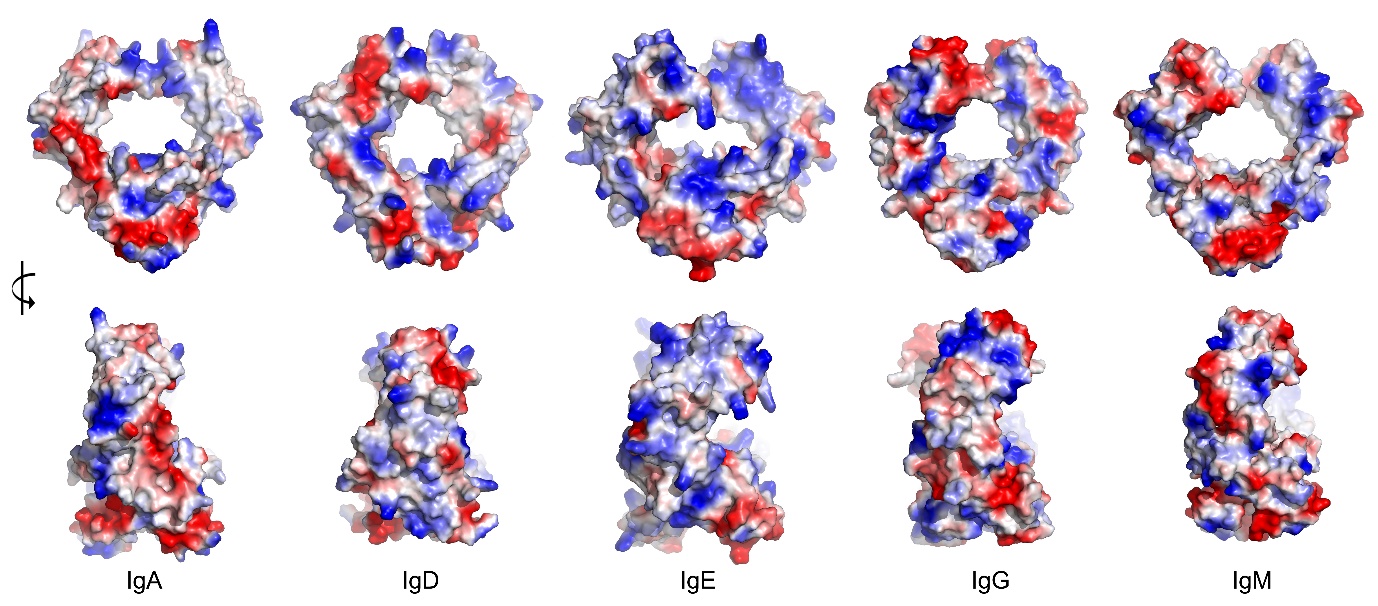


**Figure S3.** Electrostatic potential of human antibody isotypes. Positively charged regions are coloured in blue and negatively charged regions are coloured in red. Residues with missing atoms were modelled in Coot^6^ using standard rotamers. The Cε2 and Cμ2 domains are not shown. For IgD, chains C and D of the asymmetric unit are shown. Any missing Cδ3 domain residues that had been modelled in other chains were incorporated into the Cδ3 domains in chains C and D such that each domain contained residues Pro401-Asp413, Glu416-Pro455, Arg459-Pro474 and Gln478-Leu501. The following structures were used to generate the figure: IgA, PDB: 1OW0^1^; IgE, PDB: 5MOL^2^; IgG, PDB: 1L6X^7^; IgM, PDB: 8BPG.^8^ Surface electrostatic potentials were calculated and figures were produced with PyMOL.

**
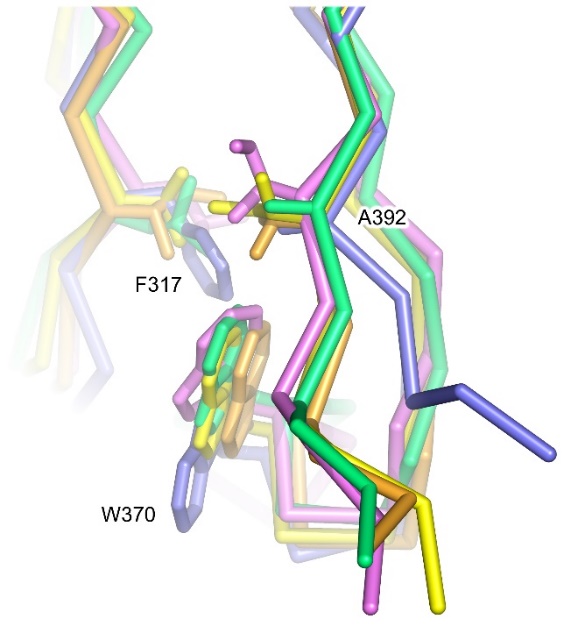
**

**Figure S4.** Conformational change in the Cδ2 domain β-strand G. In the Cδ2 domain (blue), phenylalanine is found at position 317 compared with smaller aliphatic residues in IgA (green), IgE (orange), IgG (yellow) and IgM (purple). In IgD, the W370 side chain adopts a different conformation to that found in other isotypes to avoid steric clashes with F317. In IgD, a conformational change also occurs in the A392 main chain that disrupts an interaction (which is conserved in other isotypes) between W370 and the β-strand and Cδ2-Cδ3 domain linker. The following structures were used to generate the figure: IgA, PDB: 1OW0^1^; IgE, PDB: 5MOL^2^; IgG, PDB: 1L6X^7^; IgM, PDB: 8BPG.^8^ The L338 side chain in IgA (structurally equivalent to A392 in IgD) is partially disordered.

**
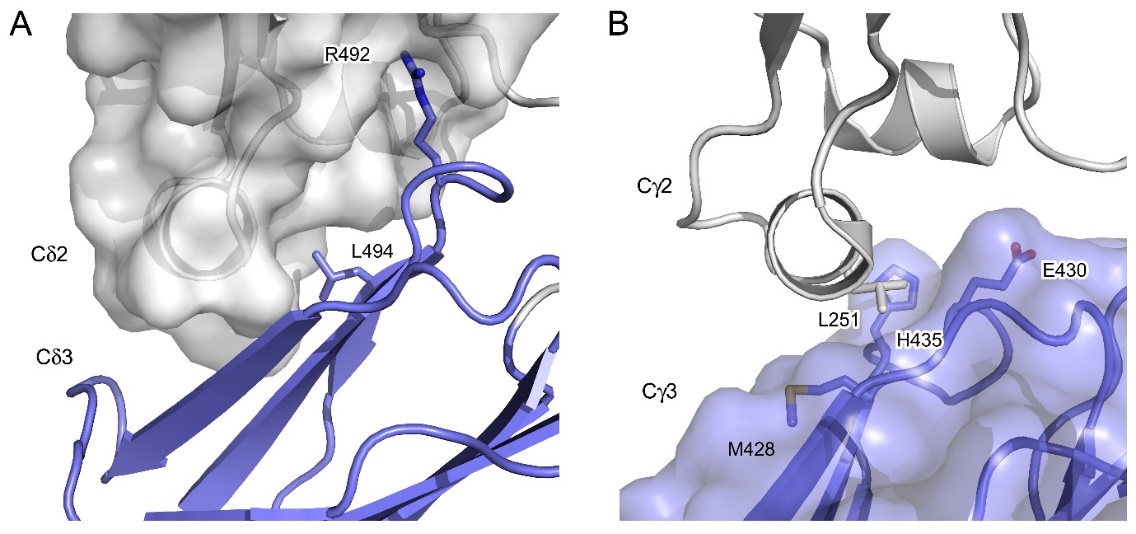
**

**Figure S5.** Pockets in IgD and IgG. (A) In IgD, two pockets on the Cδ2 domain surface (grey) accommodate R492 and L494 from the Cδ3 domain (blue). (B) In IgG, a pocket on the Cγ3 domain surface (blue) accommodates L251 from the Cγ2 domain (grey). The following structure was used to generate the figure: IgG, PDB: 4HAF.^9^

**
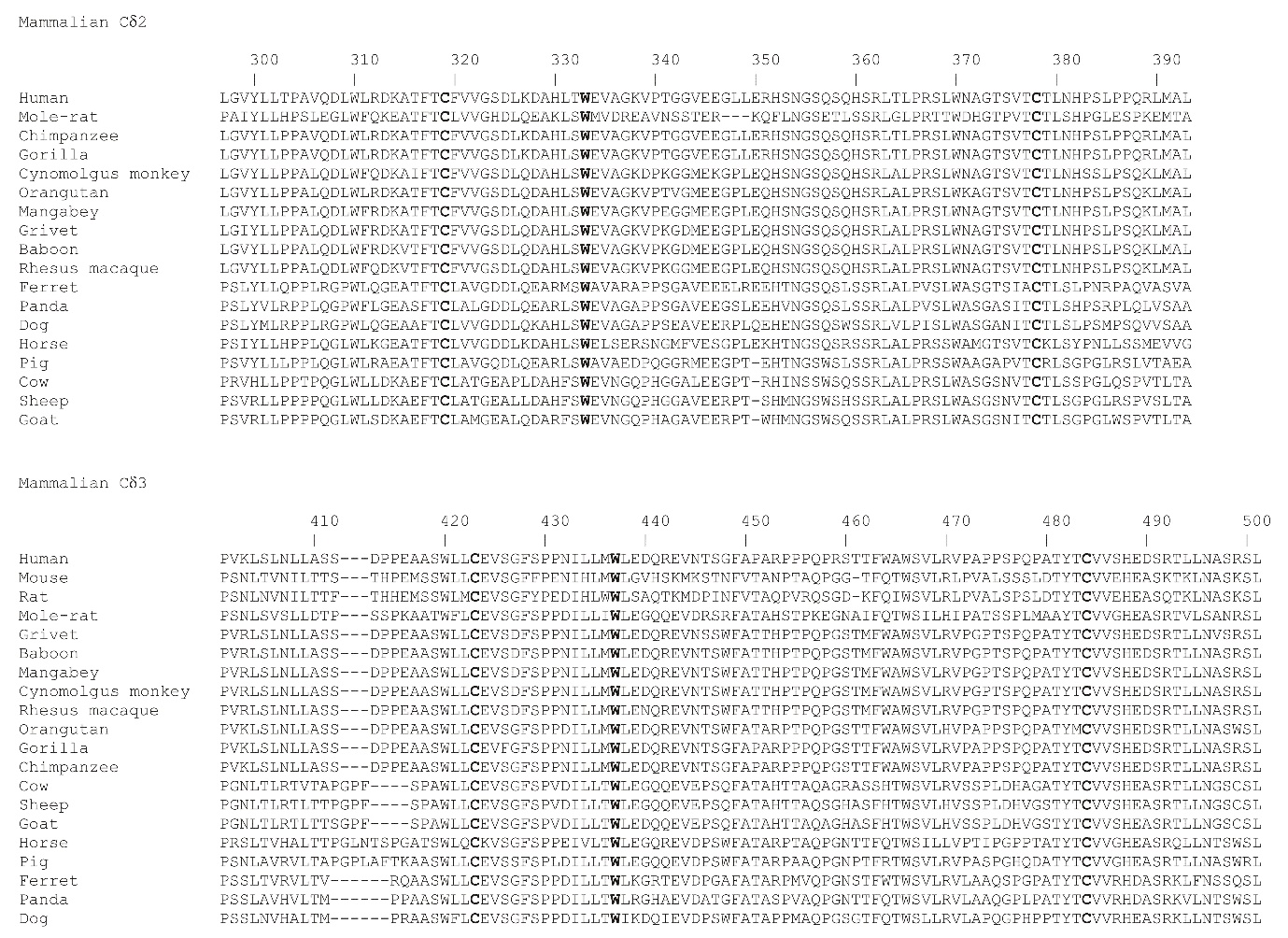
**

**Figure S6.** Sequence alignment of mammalian IgD constant domain sequences. The human IgD sequence was obtained from UniProt^10^ with accession number P01880. Mammalian IgD sequences were obtained from GenBank^11^ using the following accession numbers: baboon, ABB89458; chimpanzee, DQ297174; cow, AF411240; cynomolgus monkey, ABB89460; dog, ABB89467; ferret, QJY40730; goat, AMP34156; gorilla, DBA12041; grivet, WEL32150; horse, AAU09793; mangabey, ABB89465; mole-rat, KFO35301; mouse, AAB59654; orangutan, PNJ04969; panda, AAX73311; pig, BAI82567; rat, AAO19643; rhesus macaque, DQ297179 and sheep, AAN03671. The sequence alignments were performed with Clustal Omega^12^ and the numbering scheme used is for human IgD. The conserved amino acids of the 'central pin' residues of immunoglobulin domains^13^ are in bold.

**
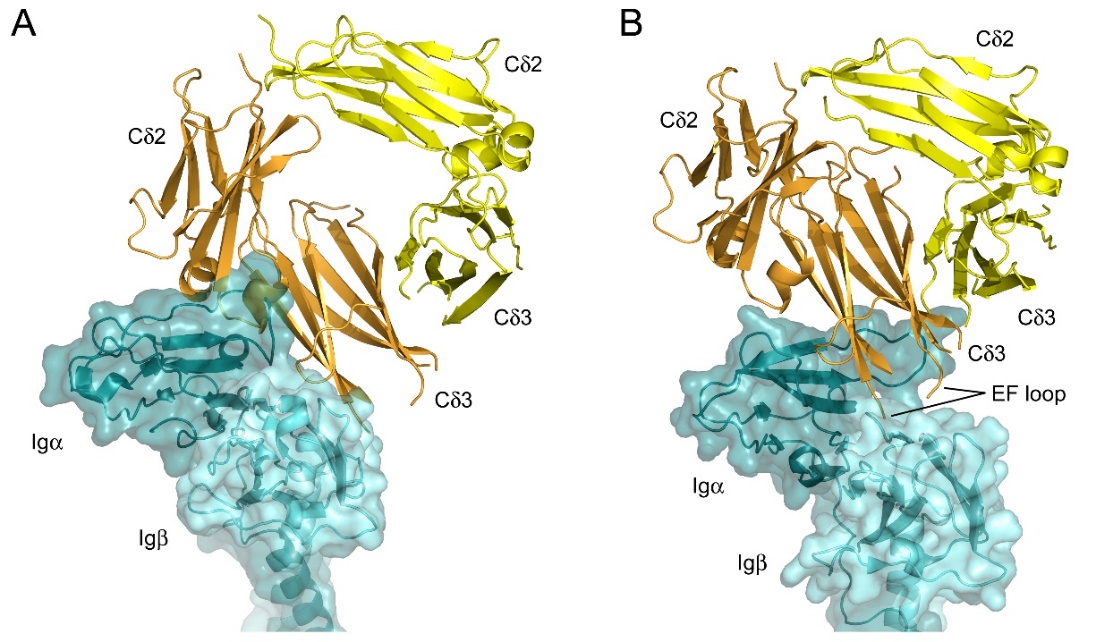
**

**Figure S7.** Superposition of the IgD-Fc structure on the IgM and IgG B-cell receptor structures. In panels (A) and (B), the IgD Cδ3 domain was superposed on the IgM Cμ4 domain and IgG Cγ3 domain that interacts with the Igα and Igβ heterodimer. For clarity, only Igα and Igβ from the B-cell receptor structures are shown and the IgM and IgG domains are hidden. In both panels, Igα and Igβ are coloured teal and cyan, respectively, and IgD-Fc is coloured orange and yellow. (A) Comparison with the IgM B-cell receptor structure^14^ reveals substantial clashes between one Cδ2 domain (orange) and Igα (teal). (B) Comparison with the IgG B-cell receptor structure^15^ reveals potential clashes between the partially disordered Cδ3 domain EF loop and Igβ. The following structures were used to generate the figure: IgM B-cell receptor, PDB: 7XQ8^14^; IgG B-cell receptor, PDB: 7WSO.^15^

**
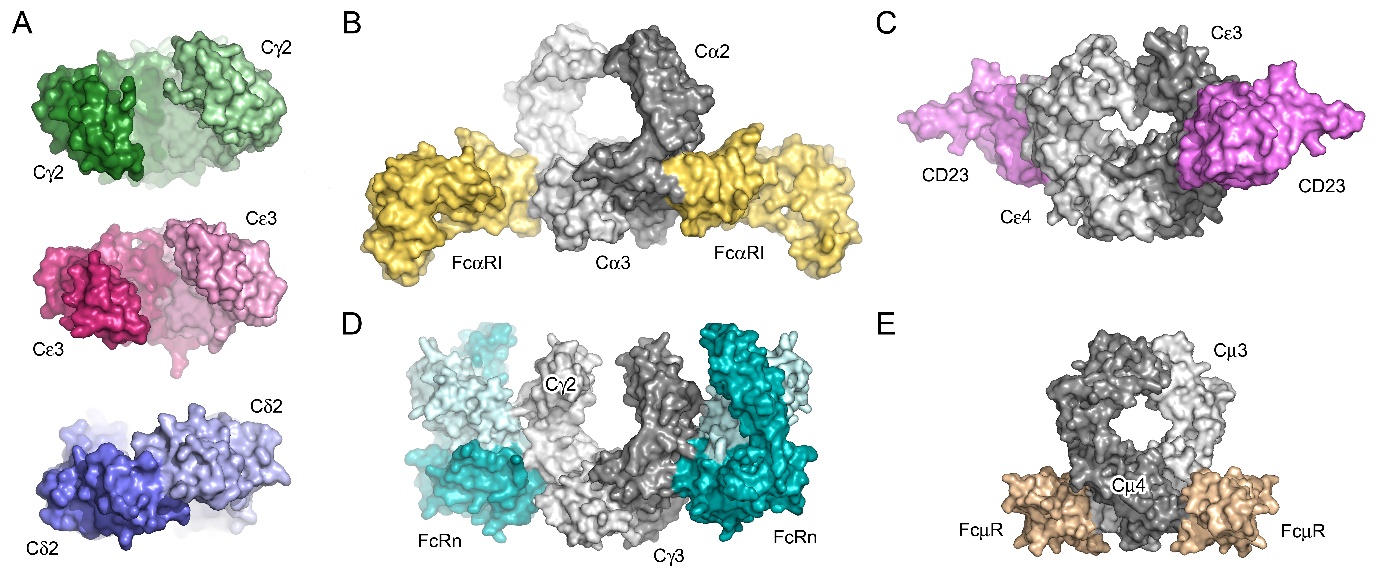
**

**Figure S8.** Receptor binding by different human antibody isotypes. (A) The Cγ2 domains in IgG (green) and the Cε3 domains in IgE (pink) adopt a similarly open conformation when they engage FcγRs (IgG) and FcεRI (IgE). The Cδ2 domains in IgD (blue) adopt a conformation that would be too closed to engage a receptor in a similar manner. For clarity, the receptors are not shown and the IgE Cε2 domains are not shown. The following structures were used to generate the figure: IgG1-Fc/FcγRI complex, PDB: 4X4M^16^; IgE-Fc/FcεRI complex, PDB: 2Y7Q.^17^ Chains A and B of the IgD-Fc structure are shown. (B) IgA-Fc (light and dark grey) binds FcαRI (yellow) at the Cα2/Cα3 domain interface. PDB: 1OW0.^1^ (C) The Cε3-Cε4 region in IgE-Fc (light and dark grey) binds CD23 (purple) at the Cε3/Cε4 domain interface. PDB: 4EZM.^5^ (D) IgG-Fc (light and dark grey) binds FcRn (light and dark cyan) at the Cγ2-Cγ3 domain interface. PDB: 4N0U.^18^ (E) The Cμ4 domains in IgM (light and dark grey) bind FcμR (wheat). PDB: 8BPG.^8^

**References**

1. Herr A, Ballister E, Bjorkman P. Insights into IgA-mediated immune responses from the crystal structures of human FcαRI and its complex with IgA1-Fc. *Nature.* 2003;423:614–620.

2. Doré KA, Davies AM, Drinkwater N, Beavil AJ, McDonnell JM, Sutton BJ. Thermal sensitivity and flexibility of the Cε3 domains in immunoglobulin E. *Biochim Biophys Acta, Proteins Proteomics.* 2017;1865:1336-1347.

3. Matsumiya, S, Yamaguchi Y, Saito J, Nagano M, Sasakawa H, Otaki S, Satoh M, Shitara K, Kato K. Corrigendum to “Structural Comparison of Fucosylated and Nonfucosylated Fc Fragments of Human Immunoglobulin G1” [J. Mol. Biol. 386/3 (2007) 767–779]. *J Mol Biol*. 2011;408:1001

4. Li Y, Wang G, Li N, Wang Y, Zhu Q, Chu H, Wu W, Tan Y, Yu F, Su XD, Gao N, Xiao J. Structural insights into immunoglobulin M. *Science*. 2020;367:1014-1017.

5. Dhaliwal B, Yuan D, Pang MOY, Henry AJ, Cain K, Oxbrow A, Fabiane SM, Beavil AJ, McDonnell JM, Gould HJ, Sutton BJ. Crystal structure of IgE bound to its B-cell receptor CD23 reveals a mechanism of reciprocal allosteric inhibition with high affinity receptor FcεRI. *Proc Natl Acad Sci* U S A. 2012;109:12686-12691.

6. Emsley P, Lohkamp B, Scott WG, Cowtan K. Features and development of Coot. *Acta Crystallogr D Biol Crystallogr*. 2019;66:486-501.

7. Idusogie EE, Presta LG, Gazzano-Santoro H, Totpal K, Wong PY, Ultsch M, Meng YG, Mulkerrin MG. Mapping of the C1q Binding Site on Rituxan, a Chimeric Antibody with a Human IgG1 Fc. *J Immunol.* 2000;164:4178–4184.

8. Chen Q, Menon RP, Masino L, Tolar P, Rosenthal PB. Structural basis for Fc receptor recognition of immunoglobulin M. *Nat Struct Mol Biol*. 2023;30:1033-1039.

9. Teplyakov A, Zhao Y, Malia TJ, Obmolova G, Gilliland GL. IgG2 Fc structure and the dynamic features of the IgG CH_2_-CH_3_ interface. *Mol Immunol*. 2013;56:131-9.

10. UniProt Consortium. UniProt: the Universal Protein Knowledgebase in 2023. *Nucleic Acids Res*. 2023;51:D523-D531.

11. Benson DA, Cavanaugh M, Clark K, Karsch-Mizrachi I, Lipman DJ, Ostell J, Sayers EW. GenBank. *Nucleic Acids Res*. 2013;41:D36-42.

12. Madeira F, Pearce M, Tivey ARN, Basutkar P, Lee J, Edbali O, Madhusoodanan N, Kolesnikov A, Lopez R. Search and sequence analysis tools services from EMBL-EBI in 2022. *Nucleic Acids Res*. 2022;50:W276-W279.

13. Williams AF, Barclay AN. The immunoglobulin superfamily - domains for cell surface recognition. *Annu Rev Immunol*. 1988;6:381-405.

14. Su Q, Chen M, Shi Y, Zhang X, Huang G, Huang B, Liu D, Liu Z, Shi Y. Cryo-EM structure of the human IgM B cell receptor. *Science.* 2022;377:875-880.

15. Ma X, Zhu Y, Dong D, Chen Y, Wang S, Yang D, Ma Z, Zhang A, Zhang F, Guo C, Huang Z. Cryo-EM structures of two human B cell receptor isotypes. *Science.* 2022;377:880-885.

16. Lu J, Chu J, Zou Z, Hamacher NB, Rixon MW, Sun PD. Structure of FcγRI in complex with Fc reveals the importance of glycan recognition for high-affinity IgG binding. *Proc Natl Acad Sci* U S A. 2015;112:833-838.

17. Holdom MD, Davies AM, Nettleship JE, Bagby SC, Dhaliwal B, Girardi E, Hunt J, Gould HJ, Beavil AJ, McDonnell JM, Owens RJ, Sutton BJ. Conformational changes in IgE contribute to its uniquely slow dissociation rate from receptor FcεRI. *Nat Struct Mol Biol*. 2011;18:571-576.

18. Oganesyan V, Damschroder MM, Cook KE, Li Q, Gao C, Wu H, Dall'Acqua WF. Structural insights into neonatal Fc receptor-based recycling mechanisms. *J Biol Chem*. 2014;289:7812-7824.
